# Harmol hydrochloride dihydrate induces autophagy in neuro cells and promotes the degradation of α‐Syn by Atg5/Atg12‐dependent pathway

**DOI:** 10.1002/fsn3.3031

**Published:** 2022-08-12

**Authors:** Gulinuer Abulimiti, Jianghua Zeng, Mutalifu Aimaiti, Xiuying Lei, Na Mi

**Affiliations:** ^1^ Department of Translational Medicine The Affiliated Kizilsu Kirghiz Autonomous Prefecture People's Hospital of Nanjing Medical University, Artux Xinjiang China; ^2^ Central Laboratory Xinjiang Medical University Xinjiang China; ^3^ Department of Biochemistry and Molecular Biology Xinjiang Medical University Xinjiang China; ^4^ State Key Laboratory of Pathogenesis Prevention and Treatment of Central Asian High Incidence Diseases Clinical Medical Research Institute The First Affiliated Hospital of Xinjiang Medical University Xinjiang China

**Keywords:** autophagy, Harmol hydrochloride dihydrate, neuro cell, α‐Syn

## Abstract

Harmol hydrochloride dihydrate (HHD) is a novel alkaloid salt of the natural β‐carboline harmol, which is isolated from *Peganum harmala* L. Here, we studied whether HHD could induce autophagy in neuro cells and investigated the underlying molecular mechanism. After incubation with HHD, the number of GFP‐LC3 puncta in cells was measured using confocal microscopy. The distribution and colocalization of autophagosomes and autolysosomes in the cells were also detected. LC3 was gathered and cultured in a medium containing HHD. Compared with control cells and cells starved for 2 h, the number of GFP‐LC3 puncta and the LC3‐II expression level were significantly increased in HHD‐treated cells (*p* < .05). The number of autophagosome (red) was increased and most of them were colocalized with lysosomes (green). Moreover, HHD induced the formation of puncta with Lysotracker Red positive in the L3 fat bodies (*p* < .05). When treated HEK cells with HHD, the protein expression level of LC3‐II was markedly increased, and the protein expression level of α‐Syn was significantly decreased (*p* < .05). HHD could induce the increased autophagosome in neuro cells by induction of autophagy. Moreover, HHD may promote the degradation of α‐Syn protein to protect neuro cells by inducing autophagy.

## Graphical abstract

This manuscript is relevant for finding that harmol hydrochloride dihydrate (HHD) could induce the increased autophagasomes in nerve cells by an induction of autophagy. Moreover, HHD may promote the degradation of ‐Syn protein to protect neuro cells by inducing autophagy.

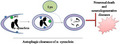

## 1 INTRODUCTION

With the aging of the population, neurodegenerative diseases have become a major threat to human health (Heemels, 2016). One of the obvious pathological features of Parkinson's disease (PD) is the accumulation of Lewy bodies in dopaminergic neurons. Lewy bodies are mainly composed of misfolded α‐Synuclein (α‐Syn) and some other proteins, such as ubiquitin proteins and p62 (Shults, 2006; von Bohlen Und Halbach, 2004). It has been reported that the aggregation of α‐Syn and the dysfunction of clearing function are closely associated with the progression of PD (Peelaerts et al., 2015). Those abnormal accumulative proteins cannot be removed in time, and their long‐term accumulation in the cytoplasm can produce neurotoxicity, which will eventually lead to the stress and apoptosis of neuro cells (Castillo‐Carranza et al., 2018). The clearance of these abnormal proteins depends on autophagy (Guo et al., 2018).

Autophagy is a conserved lysosomal degradation pathway in eukaryotic cells. It plays an important role in protein metabolism, organelle renewal, and tissue development (Mehrpour et al., 2010; Mizushima et al., 2008; Mizushima & Klionsky, 2007). The role of autophagy in neurodegenerative diseases is receiving more and more attention (Nixon, 2013). Neuronal autophagy disorder and abnormal protein aggregation are the main pathological changes in neurodegenerative diseases. Autophagy regulates the clearance of aggregated proteins in Alzheimer's disease, Parkinson's disease, and Huntington's disease, so it can ameliorate the progression of neurodegenerative diseases (Radad et al., 2015). Additionally, some natural drugs, including resveratrol, trehalose, and curcumin, play active roles in neurodegenerative diseases by inducing autophagy (Kou & Chen, 2017; Maiti & Dunbar, 2018; Rubinsztein et al., 2015).

*Peganum harmala* L. (Luo tuo peng in China) is a vital ingredient in traditional Chinese medicine for the treatment of diseases in the respiratory system, cardiovascular system, and central nervous system (Berrougui et al., 2006; Liu et al., 2015; Soliman & Fahmy, 2011). *Peganum harmala* L. contains alkaloids, steroids, flavonoids, anthraquinone, amino acids, and other components. Alkaloids are the most abundant contents with the most pharmacological activity (Wang et al., 2018). Previous studies have shown that alkaloids have anti‐inflammatory, anticardiovascular, antineurodegenerative, and other benefits (Silvestri, 2013; Tiedemann et al., 2008; Zou et al., 2017). However, there are few reports on its pharmacology. Alkaloids are insoluble in water, and it is difficult to be absorbed to participate in metabolism. Harmol hydrochloride dihydrate (HHD) is a novel alkaloid salt with low toxicity and high water solubility. In this study, we explored the effect of HHD on autophagy and investigated whether it can promote the degradation of α‐Syn through the autophagy pathway.

## 2 MATERIALS AND METHODS

### 2.1 Cell lines and reagents

NRK cells, GFP‐LC3 stable‐expressed NRK cells, Atg5 knock‐down, and Atg12 knock‐ out cells were acquired from the Laboratory of Professor Yu Li in the School of Life Sciences, Tsinghua University (Beijing, China). GN‐link‐α‐Syn and α‐Syn‐GC stable‐expressed HEK cells were the gifts from professor Jie Qiong Tan from the Central South University. Harmol hydrochloride dihydrate (HHD) powder was bought from Yuanye Biotechnology. Dulbecco's modified Eagle's medium (DMEM) was purchased from Hyclone. Fetal bovine serum (FBS) was purchased from Industries (Beit HaEmek). Dulbecco's phosphate‐buffered saline (DPBS) was bought from Gibco. Rapamycin (Rapa), bafilomycin A1 (BafA1), and 3‐methyladenie (3‐MA) were obtained from Sigma‐Aldrich. Anti‐LC3‐II, anti‐p62, and anti‐β‐actin primary antibodies were from Sigma‐Aldrich. Anti‐p‐P70s6k and anti‐p‐AMPK primary antibodies were purchased from Cell Signaling Technology. HRP‐labeled goat, anti‐rabbit IgG secondary antibody was from Southern Biotech. ECL chemiluminescence reagents were the product of BOSTER.

### 2.2 Cell culture and harmol hydrochloride treatment

NRK cells were cultured in DMEM with 10% FBS, 100 U/ml streptomycin sulfate, and 100 U/ml penicillin at 37°C in a humidified atmosphere containing 5% CO_2_. HHD was dissolved in dimethyl sulfoxide (DMSO) to be the stock solution (10 mg/ml), and then it was diluted to effective working concentrations with a culture medium. Cells were treated with harmol hydrochloride, with effective working concentrations of 10 mg/L.

### 2.3 Immunofluorescence and transmission electron microscopy analysis

Cells were fixed in 4% paraformaldehyde and blocked for 30 min with 2% BSA. After being incubated with the primary antibody overnight at 4°C and the secondary antibody for 1 h at room temperature, the stained cells were observed under a confocal microscope using a filter set for Alexa 488 and Alexa 546. After being fixed in 2.5% glutaraldehyde for 1 h, the cells were paved into cell sections and visualized with transmission electron microscopy.

### 2.4 Western blot analysis

Cells were lysed using RIPA lysis buffer (Beyotime Institute of Biotechnology) containing protease inhibitor and phosphatase inhibitor (Sangon Biotech Inc.). Protein concentrations were measured by the BCA assay (Beyotime Institute of Biotechnology). The equal amounts of proteins (20 μg) were separated by SDS‐PAGE and then transferred onto NC membrane. Membranes were blocked in 5% dried skimmed milk dissolved in phosphate‐buffered saline (PBS) and Tween‐20 (PBST) for 1 h. Then, membranes were incubated with the specific primary antibodies and respective secondary antibodies. After washing three times with TBST, the signals were detected using ECL chemiluminescence reagents (Tsea biotech Co.). Molecular Imager Chemi Doc XRS (BIO‐RAD Co.) and JS‐780 automatic gel imaging analysis systems were used for blotting and quantitative analysis.

### 2.5 Drosophila culture and drug feeding

Drosophila was fed at 25°C and 75% humidity on standard corn (20%) meal medium and sucrose (10%) supplemented with dry yeast (1.5%). HHD was initially dissolved in DMSO and then diluted in water to its effective working concentration. For the L3 larvae, we prepared the drug containing water added into sucrose (20%) and dry yeast (1.5%). In the control group and starvation group, sucrose (2%) and the same amount of DMSO were mixed into diluted water. L3 larvae were transferred to the drug‐containing medium or starvation medium for incubation.

### 2.6 Lysotracker staining and quantitative analysis of autophagic structures

L3 larvae were separated using fine forceps under a stereomicroscope. After exposure to the incubation solution and staining, the fat body was stained in 250 nM of LysoTracker red in PBS for 20 min at room temperature. Then, the larvae carcasses were washed once in PBS and transferred to a glass slide and the rest of the organization was discarded. We observed the fat body lobes covered with a cover slide by a confocal microscope and immediately observed under a standard fluorescence microscope. We obtained at least nine fat body lobes from six independent animals in each group. We randomly collected 20 fluorescent image fields to quantify the numbers of LysoTracker red‐positive spots.

### 2.7 Statistical analysis

All experiments were repeated three times with the same sample. Statistical analysis was performed by software SPSS 26.0 (International Business Machines, corp.). Significant differences between groups were assessed by one‐way analysis of variance (ANOVA). All data were expressed as means ± standard deviation (SD). *p* < .05 was considered statistically significant.

## 3 RESULTS

### 3.1 HHD induced autophagy in vitro

**FIGURE 1:**
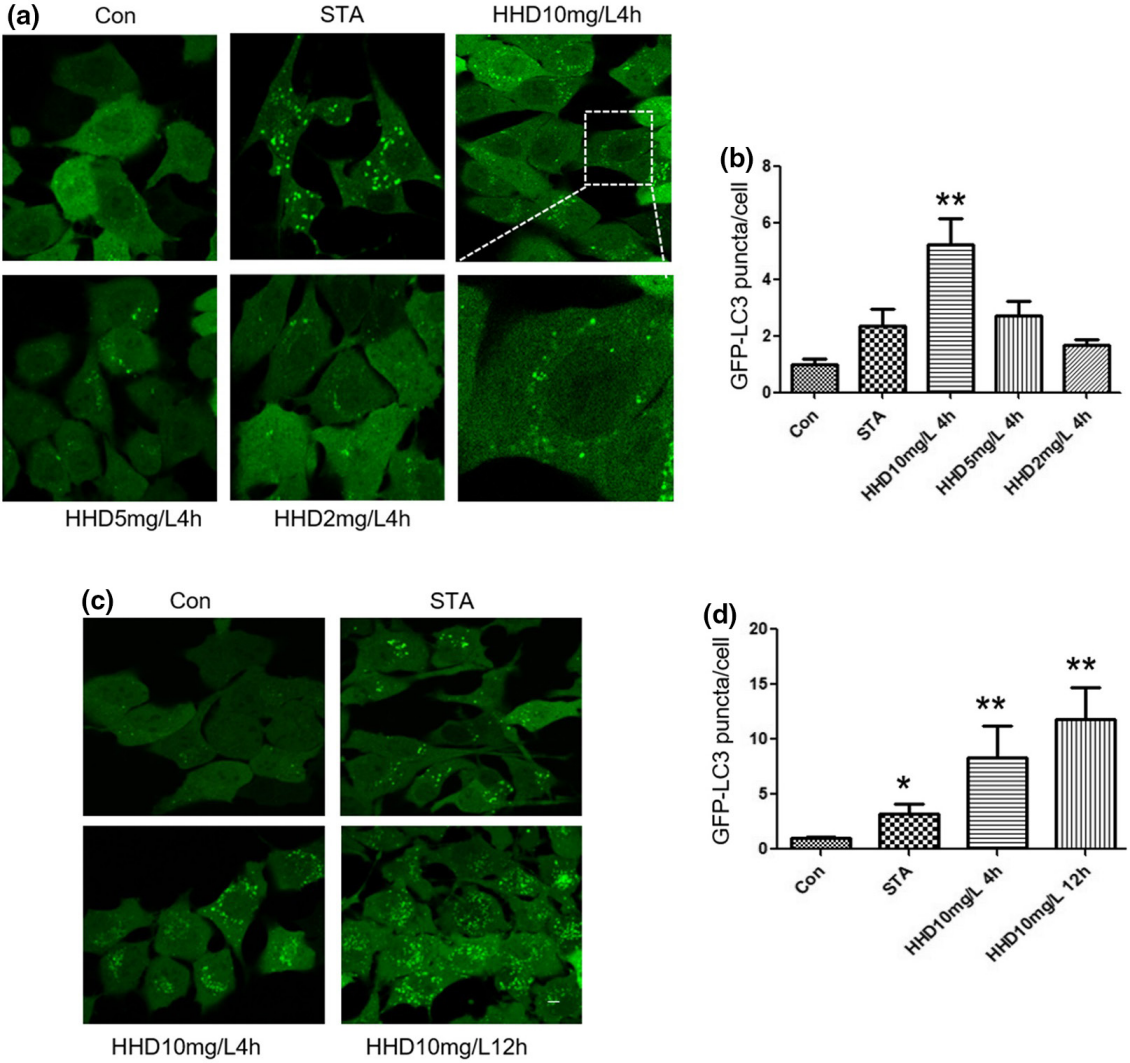
Different concentrations of HHD‐treated neuro cells. (a) GFP‐LC3‐ expressing NRK cells were cultured in DMEM supplemented with 10% FBS (control), DPBS (starvation for 2 h), and HHD (10, 5 and 2 mg/L) for 4 h. (b) Quantification of the Number of GFP‐LC3 puncta. (c) GFP‐LC3‐expressing NRK cells were cultured in DMEM supplemented with 10% FBS (control), DPBS (starvation for 2 h), and HHD (10 mg/L) for 4 or 12 h. (d) Quantification of the number of GFP‐LC3 puncta. Scale bars in full panels correspond to 10 mm. *p < .05, **p < .01, statistical analysis was carried out by Student's *t*‐test and Tukey test. DMEM, Dulbecco's modified Eagle's medium; DPBS, Dulbecco's phosphate‐buffered saline; FBS, Fetal bovine serum; HHD, Harmol hydrochloride dihydrate.

As the autophagic membrane protein, LC3‐II and P62 are important markers in autophagy levels (Rubinsztein et al., 2015; Silvestri, 2013). We measured the number of GFP‐LC3 puncta in NRK cells under confocal microscopy. The NRK cells were starved for 2 h followed by treatment with different concentrations of HHD for different times. As shown in Figure 1a,b, the number of GFP‐LC3 puncta in HHD 10 mg/L group was significantly increased compared with other groups (*p* < .01). Moreover, the number of GFP‐LC3 puncta in the HHD 10 mg/L 12 h group was significantly increased compared with HHD 10 mg/L 4 h group (*p* < .01, Figure 1c,d). Therefore, 10 mg/L HHD treated for 4 h was used for subsequent experiments.

**FIGURE 2:**
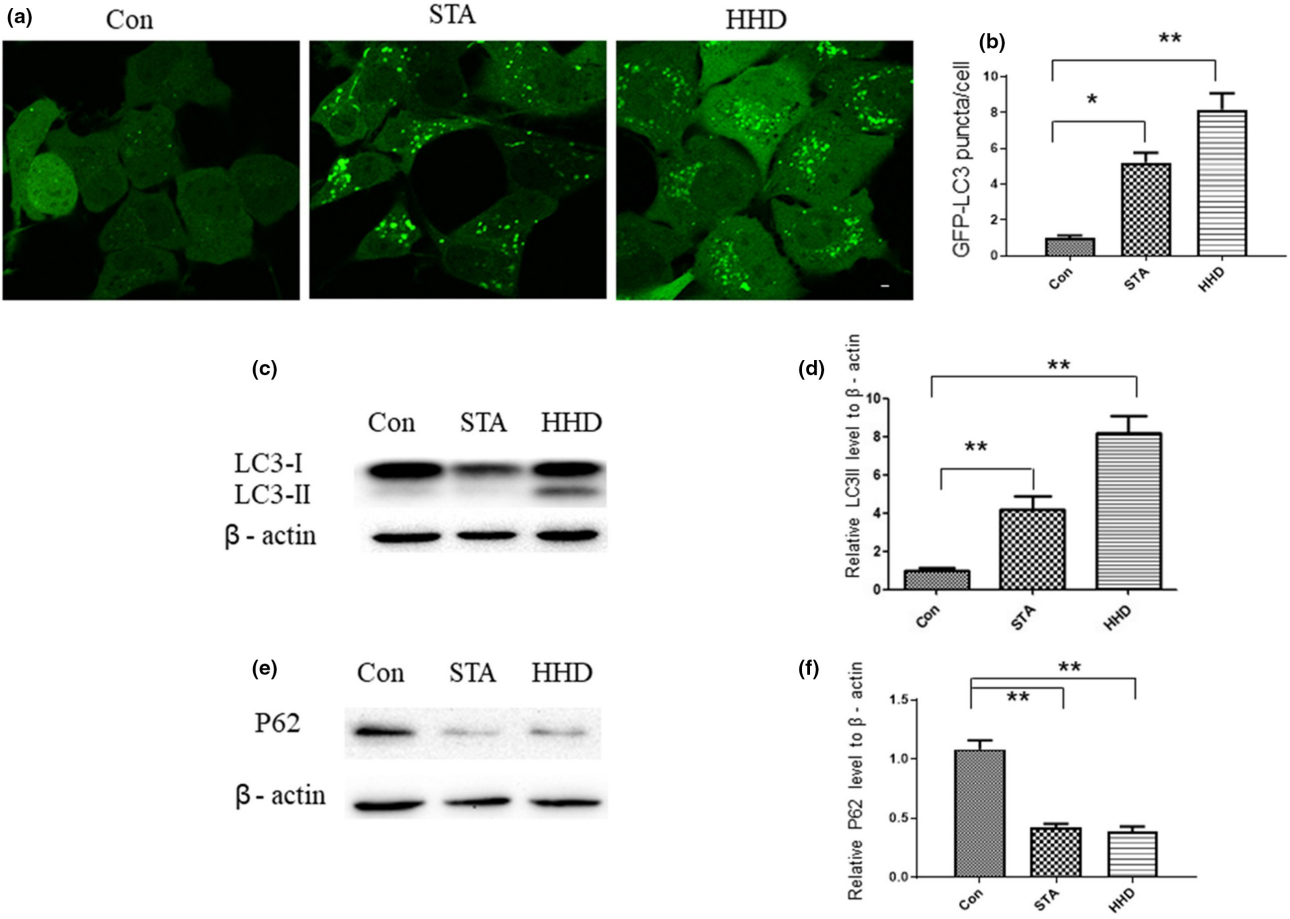
HHD induced autophagy in vitro. (a) GFP‐LC3‐expressing NRK cells were cultured in DMEM supplemented with 10% FBS (control), DPBS (starvation for 2 h), and HHD. Scale bars in full panels correspond to 10 mm. (b) Quantification of the number of GFP‐LC3 puncta. (c, e) Western blotting of LC3II and p62. (d, f) Quantification of the relative protein expression levels of LC3II and p62. *p < .05, **p < .01, statistical analysis was carried out by Student's *t*‐test and Tukey test. DMEM, Dulbecco's modified Eagle's medium; DPBS, Dulbecco's phosphate‐buffered saline; FBS, Fetal bovine serum; HHD, Harmol hydrochloride dihydrate.

Compared with control cells and starved cells, the number of GFP‐LC3 puncta was dramatically increased in HHD‐treated cells (*p* < .01, Figure 2a,b). To further verify, the protein expression level of LC3 and P62 were detected by western blotting. The data showed that the LC3‐II level was significantly increased and the P62 level was decreased in HHD‐treated group (*p* < .01, Figure 1b–f). The LC3‐II expression in HHD‐treated cells was visibly detected and data suggested that HHD could induce autophagy in vitro.

### 3.2 Autophagosome–lysosome fusion was not affected by HHD

**FIGURE 3:**
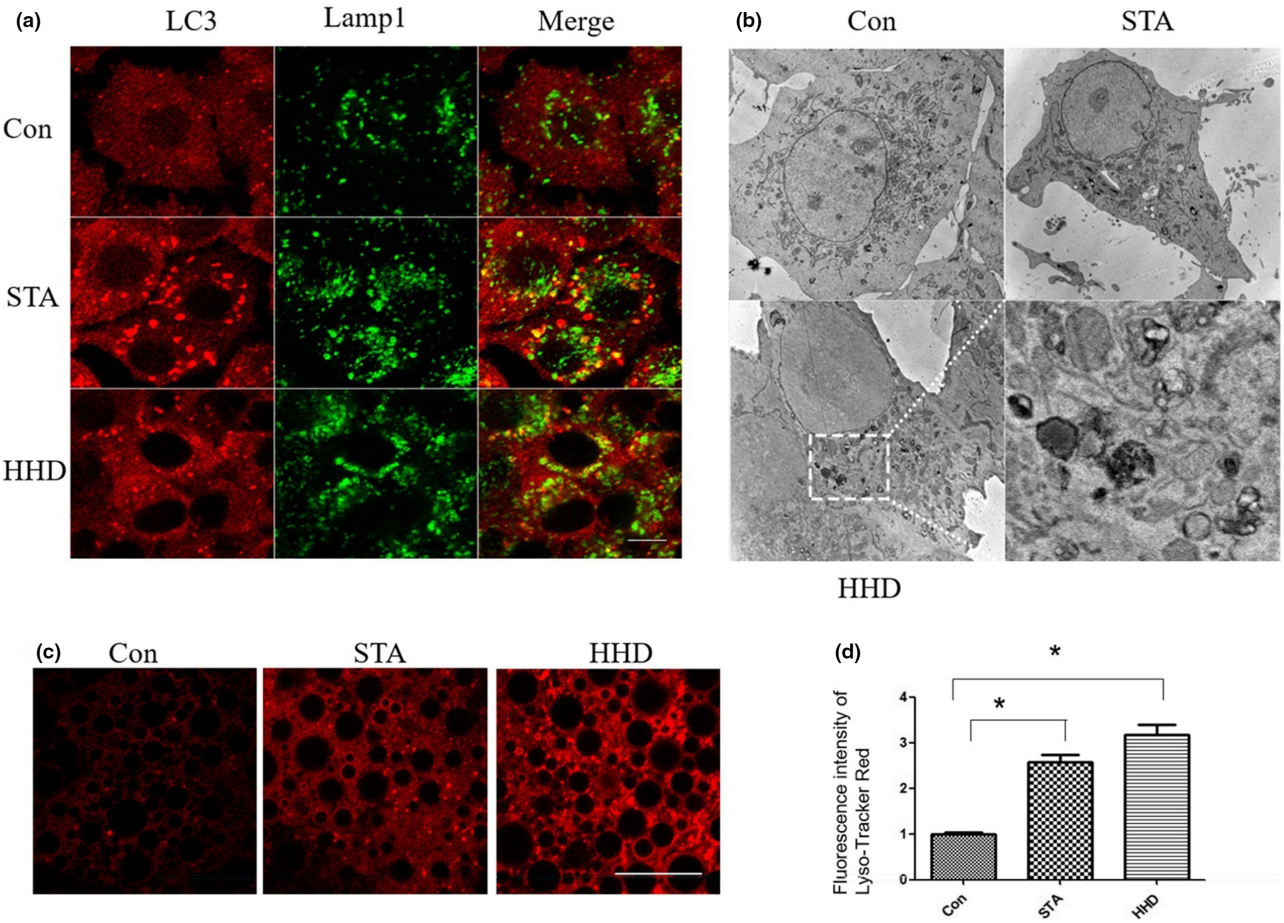
Autophagosome–lysosome fusion was not affected by HHD. (a) NRK cells were treated with HHD, and both LC3 and Lamp1 were observed by immunofluorescence. Scale bars in full panels correspond to 10 mm. (b) NRK cells with HHD were analyzed by transmission electron microscopy. Scale bars in full panels correspond to 2 μm. (c) After egg laying for 96 h, L3 larvae were gathered and fed with a culture medium containing HHD for 6 h, and fat bodies were then isolated for LysoTracker red staining. (d) Quantification of lysotracker‐positive spots in each group. *p < .05, statistical analysis was carried out by Student's *t*‐test and Tukey test. DMEM, Dulbecco's modified Eagle's medium; DPBS, Dulbecco's phosphate‐buffered saline; FBS, Fetal bovine serum; HHD, Harmol hydrochloride dihydrate.

Autolysosomes were acidified rapidly by lysosomes, which caused the content degradation (Silvestri, 2013). In order to detect the effects of HHD on autophagosome–lysosome fusion, we were able to simultaneously observe both LC3 and Lamp1 through immunofluorescence assay. After incubation with HHD, the number of autophagosomes (green) was increased, and most of them were colocalized with lysosomes (red) in comparison with the control cells (Figure 3a). After being treated with HHD, the autophagic structure was increased in HHD‐treated cells, and most structures were autolysosomes (Figure 3b). Those outcomes confirmed that the increased number of autophagosomes by HHD was caused by the induction of autophagy rather than the prevention of autophagosome degradation. To study whether HHD can induce autophagy in vivo, drosophila three instar larvae (after egg laying for 96 h) were fed with HHD, and fat bodies were then isolated for LysoTracker Red staining. As a positive control, L3 larvae were either starved for 4 h to induce autophagy. The intensity of LysoTracker Red indicated the autophagy levels and activities of the lysosome. The results showed that HHD induced the formation of LysoTracker Red‐positive puncta in the L3 larvae fat bodies, which had a similar pattern to that of the starvation group (Figure 3c,d). The data suggested that HHD could induce autophagy and enhance lysosomal activities in vivo.

### 3.3 HHD could induce autophagy in neuro cells

**FIGURE 4:**
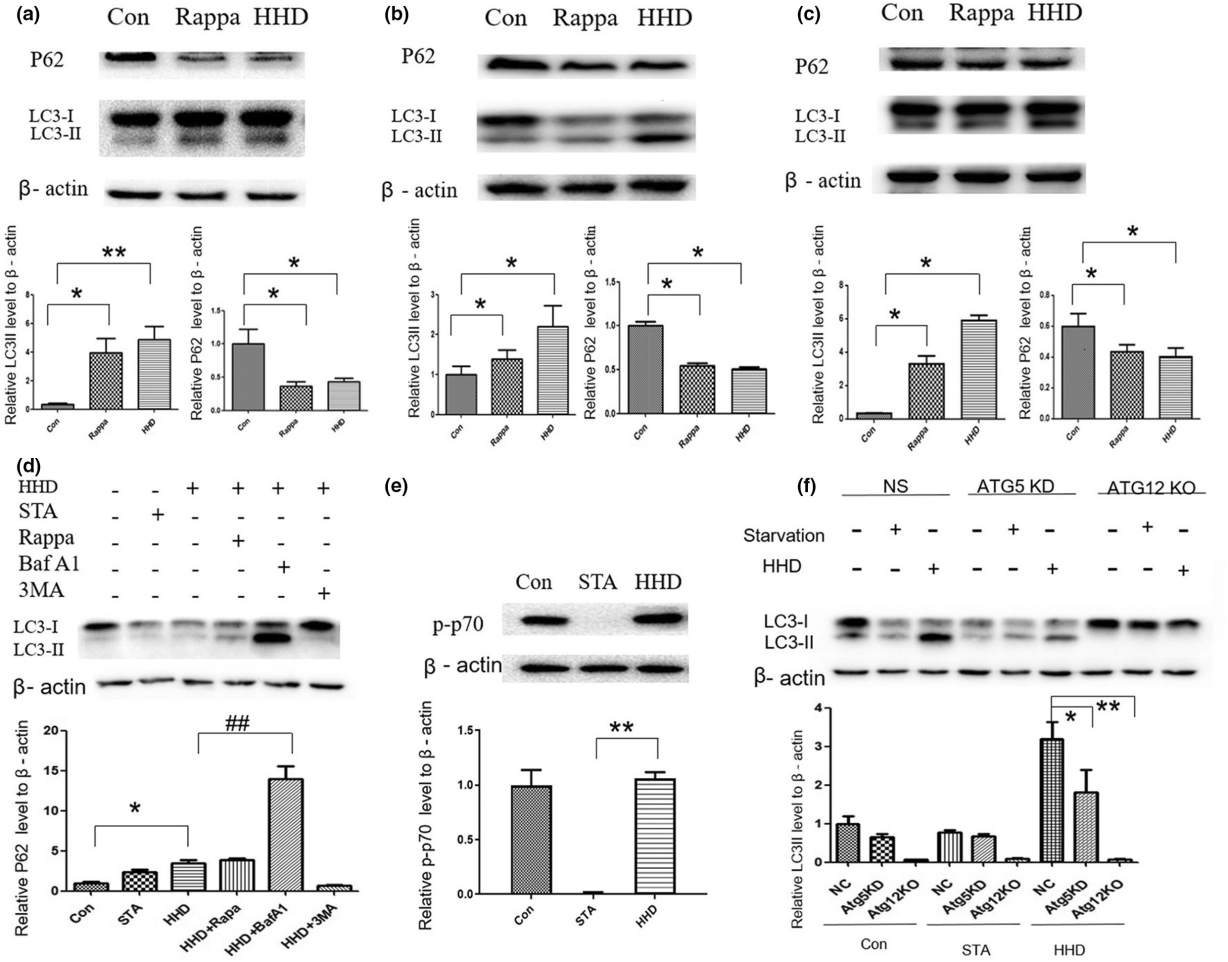
HHD could induce autophagy in neuro cell lines. (a–c) PC12SHSY‐5Y and N2a cells were treated with HHD, and western blotting was used to detect the protein levels of LC3II and p62. Quantification of the relative protein expression of p62 and LC3II. (d) Western blotting was used to detect the LC3II protein levels. Quantification of the relative LC3II expression. (e) Western blotting was used to detect the p‐p70s6k protein level. (f) Western blotting was used to detect the LC3II protein level in Atg5 knock‐down and Atg12 knock‐out cell lines. *p < .05, compared with the control group, ^##^ *p* < .01, compared with the HHD‐treated group. HHD, Harmol hydrochloride dihydrate.

Treatment with the chemical autophagy inducer rapamycin inhibits neurodegenerative changes, which inspires researchers to identify compounds that promote neuronal autophagy (Tsvetkov et al., 2010). After being treated with HHD, we detected the expression levels of LC3 and P62 in PC12, SH‐SY5Y, and N2a cell lines. LC3‐II level was dramatically increased and the degradation of P62 was evident in HHD‐treated cells (*p* < .05, Figure 4a–c). When treated with HHD and rapamycin, we found increased LC3‐II levels. When combined HHD with bafilomycin A1 (BafA1), an inhibitor of autophagosome–lysosome prevented fusion, and LC3‐II dramatically accumulated. When treated cells with HHD and 3‐methyladenine (3‐MA), the LC3‐II expression level was decreased significantly (*p* < .05, Figure 4d). The data suggested that HHD‐induced autophagy by class III phosphatidylinositol 3‐kinase (PI3K)‐dependent pathway. Furthermore, we observed the expression level of P70s6k. After starving the cells for 2 h, we discovered that the p‐p70s6k level was dramatically decreased, but there was no significant change in HHD‐treated cells (*p* < .05, Figure 4e). The data revealed that HHD may induce autophagy in an mTOR‐independent manner. Furthermore, we detected the effect of autophagy on Atg5 knock‐down and Atg12 knock‐out cells and found the LC3‐II expression level was gradually decreased, illustrating that HHD may induce autophagy through Atg5/Atg12‐dependent pathway (*p* < .05, Figure 4f).

### 3.4 HHD promoted the degradation of α‐Syn by inducing autophagy

**FIGURE 5:**
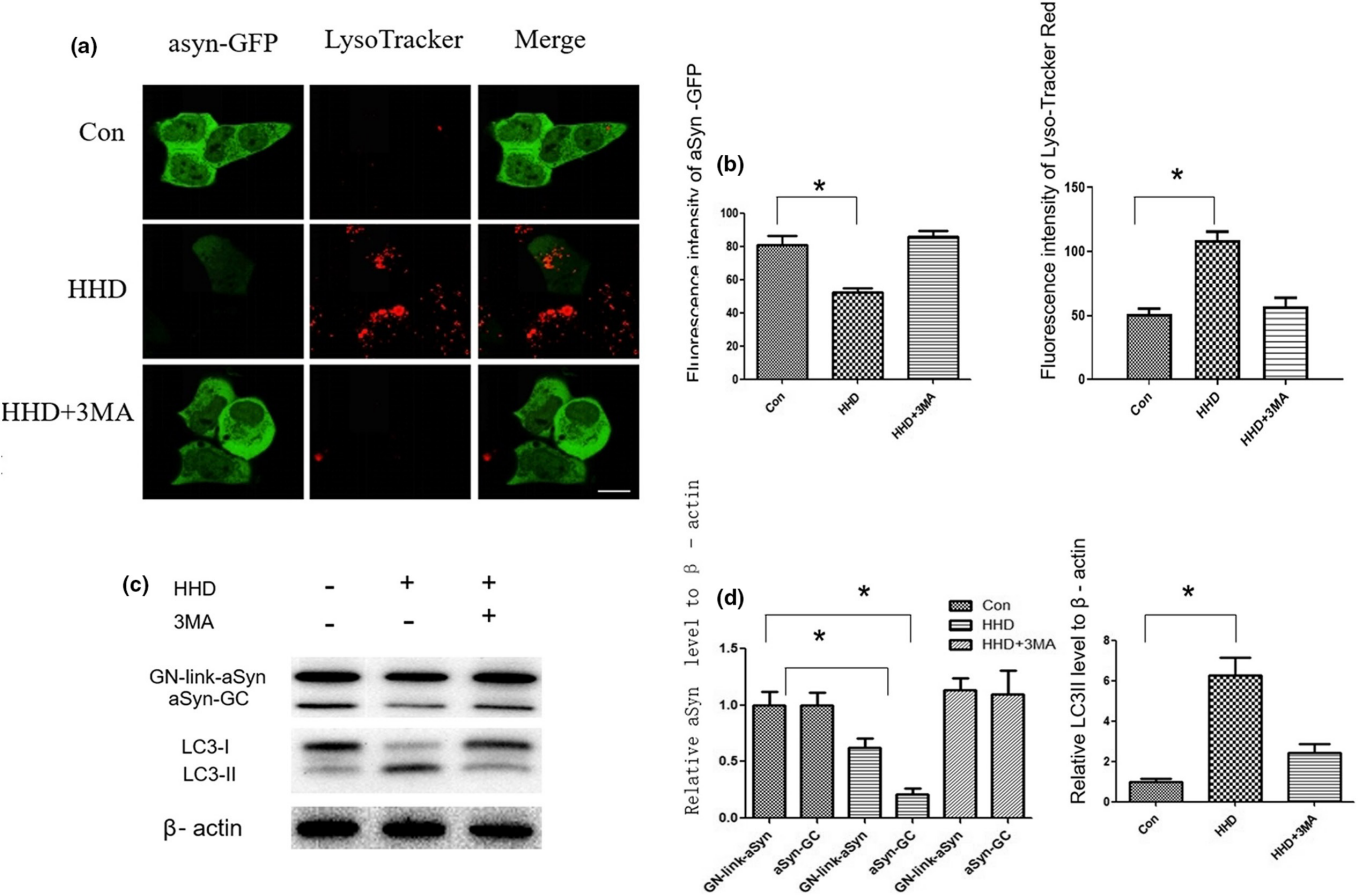
HHD promoted the degradation of α‐Syn by inducing autophagy. (a) Confocal microscopy was used to detect the fluorescence intensity of GFP. (b) Fifteen images in each group were randomly selected for fluorescence intensity analysis. (c) GN‐link‐α‐Syn and α‐Syn‐GC stable‐expressing HEK cells were treated with HHD, and western blotting was used to detect the protein levels of LC3II and α‐Syn. (d) Quantification of the relative expression of α‐Syn and LC3II. *p < .05, **p < .01. HHD, Harmol hydrochloride dihydrate.

α‐Syn is a biomarker of neurodegenerative changes in the diagnosis of Parkinson's disease. Autophagic–lysosomal pathway dysfunction is closely related to the accumulation and misfolding of α‐Syn (Bellomo et al., 2020). Autophagy is considered to be a promising therapeutic strategy to combat synucleinopathy by promoting the clearance of α‐Syn. GN‐link‐α‐Syn and α‐Syn‐GC stable‐expressing HEK cells were used in our study (Outeiro et al., 2008). To verify whether HHD could promote the degradation of α‐Syn by inducing autophagy, we treated HEK cells with HHD. The results showed that HHD could significantly reduce the fluorescence intensity of GFP and the expression of α‐Syn by inducing autophagy (Figure 5).

## 4 DISCUSSION

In this study, we reported a potent autophagy inducer, HHD, which triggered autophagic flux in a wide range of neuronal cells, and promoted the clearance of α‐Syn.

Autophagy is associated with a wide range of cellular processes, including various stresses, developmental remodeling, dynamic balance of cellular organs, and pathophysiology of the disease. It will help cells alleviate various forms of stress and contribute to monitoring the quality of organelles and proteins (Huang & Klionsky, 2007; Mehrpour et al., 2010). Some natural drugs have been shown to play active roles in neurodegenerative diseases by inducing autophagy, such as trehalose and curcumin (Maiti & Dunbar, 2018; Rubinsztein et al., 2015). This inspires researchers to identify more natural compounds that promote neuronal autophagy.

Chinese traditional medicine is beneficial to personalized therapy in disease control, bodily regulation, and health maintenance. A variety of new compounds have been used clinically, such as berberine, camptothecin, vinblastine, and matrine (Ran et al., 2017; Silvestri, 2013; Zou et al., 2017). Harmol is a β‐ carbolines (βCs) alkaloid derived from Chinese traditional medicine, *Peganum harmala* L. Alkaloids of *Peganum harmala* have neurological activity. Various in vitro and in vivo studies have shown that *Peganum harmala* and its alkaloids have effects on the central nervous system and peripheral nervous systems, such as Parkinson's disease (Leporatti & Ghedira, 2009), neurasthenia (Abu‐Irmaileh & Afifi, 2003), and analgesia (Farouk et al., 2008). The harmine extracted from *Peganum harmala* has been proved to prevent the neurotoxicity induced by overexpression of α‐Syn. However, there are few studies based on harmol in current research. HHD, as a derivative of harmol with some structural modifications, could be more easily absorbed by tissues with better solubility (Cai et al., 2019).

In this study, HHD‐treated cells showed a significant increase in its autophagic level. Compared to the control group, LC3‐II levels were significantly increased in HHD‐treated cells by the detection of autophagic flux. For in vivo experiments, we chose the fat bodies of L3 drosophila larvae that are more sensitive to nutritional changes. The results showed that HHD can induce autophagy significantly. By observing the vagueness of L3 larvae, there was no significant difference between HHD‐treated group and the control group. However, the activity of lysosomes was significantly increased.

Induction of autophagy in neuro cells is more difficult than in nonneuro cells (Tsvetkov et al., 2010). Starvation is a classical autophagy inducer, and it fails to induce autophagy in the cortex of mouse brains. The mTOR inhibitors, such as rapamycin and everolimus, induce only mild autophagy in neurons (Boland et al., 2008). Therefore, the treatment strategy based on autophagy regulation has become dawn of neurodegenerative diseases. Compared with rapamycin, we detected that the LC3‐II level was dramatically increased in PC12, SH‐SY5Y, and N2a cell lines after HHD treatment.

As an autophagy substrate, P62 is used as an indicator of autophagy activity. P62 can reflect the level of autophagy and degradation of substrates, and it was reported to colocalize with ubiquitin‐positive inclusions, such as α‐Syn. P62 plays a critical role in ubiquitinated protein degradation by linking the autophagy pathway with ubiquitin–proteasome system (Liu et al., 2016). In our findings, P62 also degraded considerably in HHD‐treated neuro cell lines, which suggested that HHD not only affected LC3 lipidation but also increased the level of autophagy. In further research, compared with HHD‐treated groups, rapamycin with HHD increased the ratio of LC3‐II. When cells were treated with both HHD and BafA1, the expression of LC3‐II was increased, indicating that HHD did not affect lysosome degradation activity. We found an extremely rare LC3‐II level in the 3‐MA group, indicating that HHD‐induced autophagy was dependent on class III PI3K activity. The mTOR is a critical factor in the regulation of autophagy. In our study, we detected the p‐p70s6k level as the downstream of the mTOR in the HHD‐treated cells. Interestingly, compared with starved cells, p‐p70s6k level in HHD‐treated cells did not decrease. It suggested that HHD‐induced autophagy might be related to mTOR‐independent pathways.

According to some recent reports, some drugs inducing autophagy by inhibiting mTOR may not be applicable clinically because mTOR inhibition can lead to immunosuppression (Schiebler et al., 2015). Neuroinflammation has been confirmed in playing a key role in the pathogenesis of neurodegenerative diseases (Chen et al., 2016). In our study, HHD‐induced autophagy by an mTOR‐independent pathway avoided the risk of drug‐related immunosuppression. Furthermore, we found that the process of LC3‐II was gradually decreased in Atg5 knock‐down and Atg12 knock‐out cells. This indicated that HHD induced autophagy by Atg5/Atg12‐dependent pathway. Atg5 and Atg12 are essential for LC3 processing. Recently, it has been reported that autophagy in regulating immune function of the central nervous system is dependent on the key autophagy regulators Atg12–Atg5 complex, revealing the role of autophagy in clearing Aβ and attenuating microglial activation (Su et al., 2016).

Different aggregation patterns of α‐Syn fibers can cause different neurodegenerative diseases. Different configurations of α‐Syn fibers are injected into the brain and blood of rats, respectively, and the results show rats with different symptoms. Cylindrical α‐Syn fibers lead to PD, while banded α‐Syn fibers cause symptoms of multiple system atrophy (MSA) (Peelaerts et al., 2015). We can infer that it may be a feasible treatment for PD to enhance the autophagy function of neurons by different means and reduce the neurodegeneration caused by α‐Syn. It has been proved that HHD can induce autophagy in neuro cell lines, so we further studied whether HHD promotes α‐Syn degradation by inducing autophagy.

We treated GN‐link‐α‐Syn and α‐Syn‐GC stable‐expressing HEK cells with HHD and the α‐Syn expression level were detected by confocal microscopy and western blotting. Compared with the control group, the fluorescence intensity of GFP was decreased after being treated with HHD. However, the fluorescence intensity of GFP did not change after being treated with 3 MA and HHD. Western blotting showed that the LC3‐II protein expression was significantly increased and the α‐Syn expression was significantly decreased after being treated with HHD. When treated HEK cells with HHD and 3 MA, autophagy was inhibited and the α‐Syn expression did not change. From the decrease in the fluorescence intensity of GFP and lower α‐Syn expression, it can be concluded that HHD could promote the degradation of α‐Syn through autophagy–lysosome pathway. Our results provide a theoretical basis for HHD to be α‐Syn potent lowering compound.

## 5 CONCLUSION

Compared with control cells and cells starved for 2 h, the number of GFP‐LC3 puncta and the LC3‐II expression level were significantly increased in HHD‐treated cells. Moreover, HHD induced the formation of puncta with Lysotracker Red positive in the L3 fat bodies. When treated HEK cells with HHD, the protein expression level of LC3‐II was markedly increased, and the protein expression level of α‐Syn was significantly decreased. HHD could induce the increased autophagosome in nerve cells by induction of autophagy and promote the degradation of α‐Syn protein to protect neuronal cells by inducing autophagy. HHD may be developed into new therapeutic agents against neurodegenerative diseases caused by autophagy disorder.

## FUNDING INFORMATION

This work was supported by the New Drug Research and Development Program of Local Traditional Chinese medicine and Ethnic Group Medicine of Xinjiang Uighur Autonomous Region (No. 2017‐02‐04) and Natural Science Foundation of Xinjiang Uighur Autonomous Region (No. 2017D01C412) and Natural Science Foundation of Xinjiang Uighur Autonomous Region‐Youth Fund (No. 2021D01B12).

## CONFLICT OF INTEREST

The authors declare that they have no conflict of interest.

## Data Availability

The datasets used or analyzed during the current study are available from the corresponding author on reasonable request.
